# Female mate choice in convict cichlids is transitive and consistent with a self-referent directional preference

**DOI:** 10.1186/1742-9994-10-69

**Published:** 2013-11-11

**Authors:** François-Xavier Dechaume-Moncharmont, Marine Freychet, Sébastien Motreuil, Frank Cézilly

**Affiliations:** 1 Équipe écologie évolutive, UMR CNRS 6282 Biogéosciences, Université de Bourgogne, 6 boulevard Gabriel, 21000 Dijon, France; 2 Institut Universitaire de France, France

**Keywords:** Mate choice, Rationality, Transitivity, Assortative mating, Self-referent directional preference, *Amatitlania nigrofasciata*

## Abstract

**Introduction:**

One of the most important decisions that an animal has to make in its life is choosing a mate. Although most studies in sexual selection assume that mate choice is rational, this assumption has not been tested seriously. A crucial component of rationality is that animals exhibit transitive choices: if an individual prefers option A over B, and B over C, then it also prefers A over C.

**Results:**

We assessed transitivity in mate choice: 40 female convict cichlids had to make a series of binary choices between males of varying size. Ninety percent of females showed transitive choices. The mean preference index was significantly higher when a female chose between their most preferred and least preferred male (male 1 vs. male 3) compared to when they chose between males of adjacent ranks (1 vs. 2 or 2 vs. 3). The results are consistent with a simple underlying preference function leading to transitive choice: females preferred males about one third larger than themselves. This rule of thumb correctly predicted which male was preferred in 67% of the cases and the ordering in binary choices in 78% of cases.

**Conclusions:**

This study provides the first evidence for strong stochastic transitivity in a context of mate choice. The females exhibited ordinal preferences and the direction and magnitude of these preferences could be predicted from a simple rule. The females do not necessarily compare two males to choose the best; it is sufficient to use a self-referent evaluation. Such a simple decision rule has important implications for the evolution of the mating strategies and it is consistent with patterns of assortative mating repeatedly observed at population level.

## Introduction

A crucial question about adaptive mate choice is to what extent individuals show some level of rationality when choosing a mate [1-4]. Assuming that natural selection has shaped the preference functions of animals, mate choice is expected to maximize fitness [5-7]. Ideally, then, animals should be able to assign to each potential mate a value on a single dimension directly related to fitness, and their probability of choosing one potential mate over another should be a monotonic function of their respective values.

Rationality in choice is traditionally equated in behavioural ecology with consistency across time, transitivity and independence from irrelevant alternatives [1,8]. Although the repeatability of mating preferences [9] and, to a lesser extent, independence from irrelevant alternatives [10,11] has been investigated in several species, analyses of transitivity remain scarce in the context of mate choice. The only previous experimental study assessed transitivity in female preference for male calls in the Tungara frog, *Physalaemus pustulosus*[12-14]. This study was inconclusive, however, as there was no sufficient evidence for either transitivity or intransitivity in mate choice. Yet, transitivity is arguably the most fundamental axiom of rational choice [15,16], and has been previously investigated in animal species in various other contexts, such as foraging decisions [17-20], nest choice [21,22] or dominance relationships [23,24].

Transitivity in choice, in its simplest definition [25], states that if an individual prefers option A over B and B over C, then it also prefers A over C. However, the level of expression of transitivity depends on whether options can be ranked on an ordinal or an interval scale [26]. Consider three alternative mating options A, B, and C. Let P(A,B) be the probability of choosing option A from the choice set {A,B}. Choice proportions conform to weak stochastic transitivity (WST) if when P(A,B) ≥ 0.5 and P(B,C) ≥ 0.5, then P(A,C) ≥ 0.5. Evidence for WST validates only the assumption of one-dimensional choice, meaning that the options can be ordered, but not quantified, on a common scale. By contrast, strong stochastic transitivity (SST) occurs if P(A,C) ≥ max{P(A,B), P(B,C)} [19,27-29], and would be indicative that not only ordinal preference holds, but that a value on a common quantitative scale can be assigned to each mating option [26].

Here, we assess transitivity in choice in relation to mating patterns in female convict cichlids, *Amatitlania nigrofasciata*, which is a model species in mate choice [30-33]. In this monogamous and territorial fish species with bi-parental care, females generally benefit from mating with a large male, in terms of breeding success [34] and protection of offspring against predators or competitors [35]. However, large males in this species are particularly aggressive, and females must weigh the benefits of pairing with a large male against negative fitness effects linked to asymmetric aggressive interactions [36] and increased risk of filial cannibalism [37,38].

Preference for partner size is thus expected to be finely tuned in this species. Previous studies have established that males show a preference for gravid, large females [39], whereas the preference of females in relation to male size remains controversial. Depending on the study, females either preferred larger males [30,40-42], or males of similar size to themselves [43]. Typically, female preference was assessed by simultaneously confronting a focal female with two [30,42] or three males [44] differing in size. Preference is then inferred from the relative amount of time spent next to each male. A female is thus assumed to prefer male A over male B (A > B) when the amount of time spent in front of male A divided by the total amount of time spent in the two choice areas, considered hereafter as the preference index, is above 50%. This measure has been shown to correlate with female reproductive preference in convict cichlids [42] and other cichlid fish [45]. No study, however, has examined transitivity in female choice when confronted with several males.

Most theory predicts that violation of rationality will only occur when the available options differ in multiple dimensions ([46,47] but see [48]). All previous experimental studies of female choice in convict cichlids consisted in confronted females with live males of different sizes and recorded their behaviour and time spent in front of each male (review in [42]). However, in addition to size, males may vary in respect to several other phenotypic attributes, such that female choice might not necessarily be one-dimensional. One way to confirm that females indeed prioritize one dimension such as body size is to show that female choice in relation to that dimension is effectively transitive. That is to say that rational, transitive female choice should not be assumed a priori, but assessed experimentally. Beyond the assessment of rationality in mate choice, analysis of transitivity may potentially help to identify the decision rules underlying mate choice and mating patterns.

**Figure 1:**
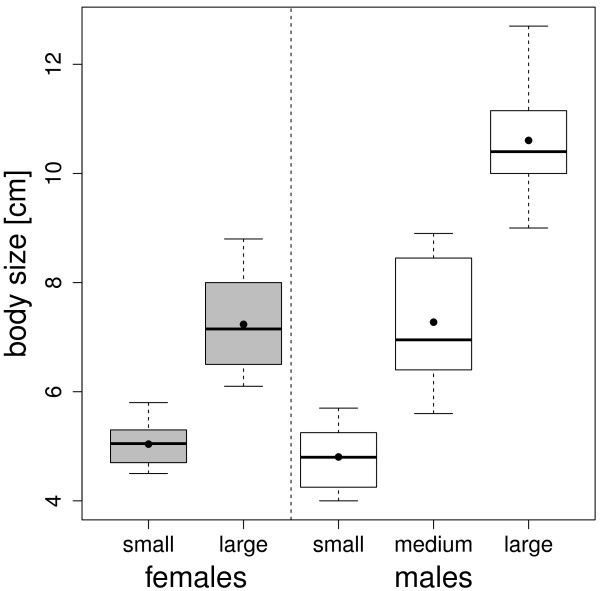
**Boxplot of the body sizes as a function of the size classes for each sex.** Body size was measured as the standard length, from the tip of the mouth to the caudal peduncle. Females (grey boxes) were assigned to two size classes: small (n = 20) and large (n = 20). Males (white boxes) were assigned to three size classes: small (n = 20), medium (n = 20), and large (n = 20). For a given size class, the black dot depicts the mean, the thick line the median, and the box the interquartile range. Within a pair of females or within a triplet of males, there was a difference of at least 1 cm between each individual.

In a sequence of binary choices, 40 females were confronted with dyads of males differing in size. We formed 20 triplets of males, consisting of one small male (S), one medium size male (M), and one large male (L). Each triplet of males was assigned to one pair of females consisting of one large and one small female (Figure 1). Each female was sequentially presented to one of the three possible male dyads (L *vs.* M, M *vs*. S, and L *v*s. S). For each female, we determined her preference ranking of the males according to pairwise comparisons over the three trials, based on a preference index above 50%. Our results are qualitatively unchanged if we use a stricter criterion of preference index larger than 55% (see Additional file 1). We show that both large and small female convict cichlids show significant SST when choosing between males of different size. In addition, we show that a simple self-referent directional rule, assuming that females prefer males which are about 30% larger than themselves, performs particularly well at predicting observed transitive choices.

## Results

### Transitivity

Females exhibited marked preferences, as the mean proportion of time spent in front of the preferred male was 71.5% (bootstrapped 95% CI = 68% to 74%). This mean preference index was not correlated between the large and small females from the same pair (Pearson correlation coefficient r = 0.11 with 95% CI = -0.35 to 0.53, n =20, p = 0.64). The mean proportion of total time spent in the no-choice (neutral) area was 12% (95% CI = 10% to 14%) and was not correlated between the large and small females from the same pair (r = 0.16 with 95% CI = -0.30 to 0.52, n = 20, p = 0.49).

Based on preference index larger than 50%, we observed transitive mate choice for 19 out of 20 large females and 17 out of 20 small ones (Table 1), with no difference between these two groups (Fisher exact test, *P* = 0.60). Overall, transitivity was significantly more frequent than expected by chance (exact binomial test B(40, 0.75), p = 0.016). Intransitivity could not be linked to a given triplet of males: intransitive females belonged to different pairs and, thus, they were presented to different triplets of males. Intransitivity could not be explained by a less decisive female behaviour either. There was no evidence that intransitive females made less pronounced choices than transitive ones, as both the preference index (F = 0.83, df = 1 and 38, p = 0.37) and the time spent in the neutral area (F = 0.45, df = 1 and 38, p = 0.51) did not differ between the two groups.

**Table 1:** Number of transitive or intransitive choices as a function of the size of the female

	**Transitive order**						**Intransitive order**	**Total**
Female size	L>M>S	L>S>M	M>L>S	M>S>L	S>L>M	S>M>L	L>M>S>L	
Small	2	1	3	5	1	7	1	20
Large	8	2	3	3	0	1	3	20
Total	10	3	6	8	1	8	4	40

Notation L > M > S reflects transitive ordering in which a female preferred the larger male over the medium size male (L > M), the medium size male over the smaller one (M > S), and the larger male over the smaller one (L > S). Four intransitive females exhibited the circular triad: L > M, M > S, and S > L.

**Figure 2:**
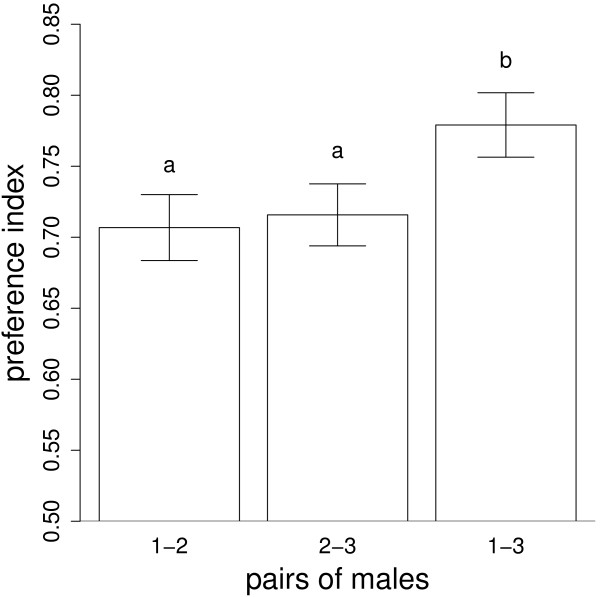
**Mean proportion of time (+/- standard error) spent on the side of the preferred male.** These preference indexes were computed for the 36 females having made a transitive ordering of the triplet of males (male 1 was preferred over male 2, male 2 over male 3, and male 1 over male 3). For example, the first bar (1–2) depicts the proportion of time spent on the side of the male 1 when tested with the male 2. Different letters mean statistically significant differences.

An overall comparison revealed that the preference indexes of the 36 transitive females strongly differed between the three dyads of males (Figure 2, F = 8.16, df = 2 and 70, p = 0.0007). Mean preference index was significantly higher when the females had to choose between males of distant ranks (male 1 vs. male 3, *i.e.* their most preferred and least preferred males), compared to when choosing between males of adjacent ranks (male 1 vs. male 2, p = 0.0028; male 2 vs. male3, p = 0.0003), whereas the preference index when choosing between male 1 vs. male 2 did not differ from that when choosing between male 2 vs. male 3 (p = 0.24). We observed a significant correlation between the preference for male 1 vs male 3 and the mean preference for male 1 vs male 2 and male 2 vs male 3 (Pearson correlation coefficient r = 0.39, 95% CI from 0.065 to 0.63, p = 0.020 ; in Additional file 1: Figure S1). The females which exhibited strong preference for male 1 vs male 2 or male 2 vs male 3 exhibited even stronger preference for male 1 vs male 3, thus providing evidence for strong stochastic transitivity in female convict cichlids.

### Self-referent preference rule

**Figure 3:**
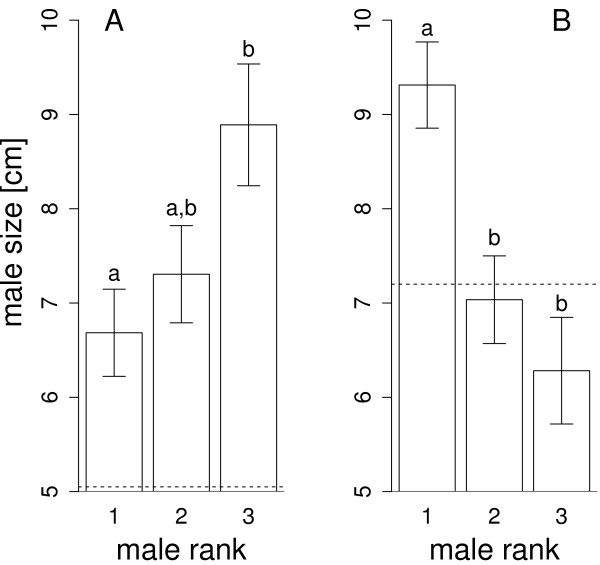
**Mean male size (+/- standard error) as a function of their rank by the transitive females.** The body size was measured as the standard length, from the tip of the mouth to the caudal peduncle. The mean male sizes were computed for the 36 females having made a transitive ordering of the triplet of males: male 1 was preferred over male 2, male 2 over male 3, and male 1 over male 3. Different letters mean statistically significant difference. The horizontal dotted line depicts the mean female size. **(A)** Choice made by the smaller females (n = 19). **(B)** Choice made by the larger females (n = 17).

According to the Condorcet criterion, the most widely accepted normative standard for rational social choice [49], we defined that size class M was socially preferred over choice alternative S (M > S) when a majority of females preferred M over S than S over M. Using such ranked pairs at the population level, our data support the social transitive ordering M > S > L. This ordering is consistent with previous observations at group level: females presented with three males of different sizes chose most often the medium-sized male [44]. However, we argue for a cautious interpretation of such pairwise majority aggregation of individual preference. It may not reflect individual preferences [50]. Indeed, mean male size decreased with rank preference for small females (Figure 3A, F = 4.11, df = 2 and 36, p = 0.024), whereas it increased for large ones (Figure 3B, F = 8.52, df = 2, 32, p = 0.001). There was no evidence for unconditional preference for either the largest male or the medium-sized one. On the contrary, the size of the most preferred male (male 1) increased with female size (F = 19.35, df = 1 and 34, p = 0.0001), potentially favouring a strong pattern of homogamy (Pearson’s correlation coefficient r = 0.6 with 95% CI = 0.34 to 0.77), consistent with that previously reported in natural populations, r = 0.57 [51].

**Figure 4:**
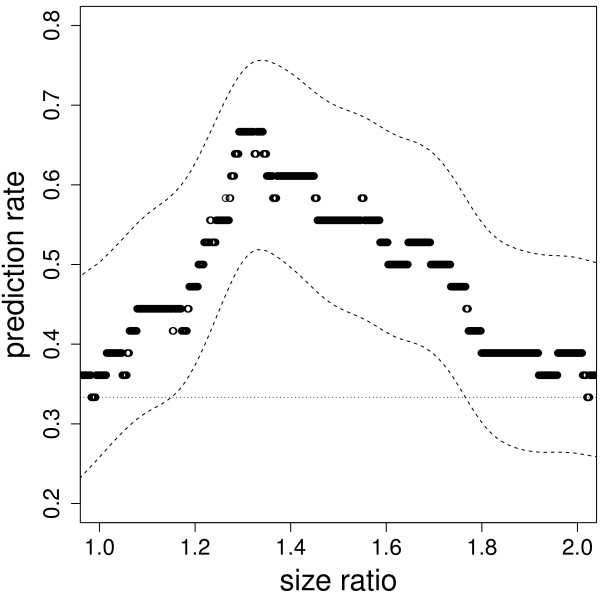
**Proportion of correct predictions of the preferred male as a function of the ideal size ratio.** For a given size ratio (λ = ideal male size/female size), a female was assumed to prefer the male leading to the ratio that was the closest to this ideal one. The dashed curves show bootstrapped 95% confidence interval around the prediction. The horizontal dotted line shows random choice (one in three chances of choosing the preferred male).

We tested the hypothesis that females showed self-referent directional preference for male size, in such a way that the preferred male size corresponded to a fixed ratio *λ* to that of the female. Most accurate predictions for male 1 were achieved for *λ* values ranging from 1.29 to 1.32 (Figure 4). With *λ* = 1.30 (i.e. females prefer males 30% larger than themselves), male 1 was successfully indentified more often than expected by chance (Table 2). This ratio also gave the best prediction rates for linear order of the three males and for choice between two males in pairwise comparison. Each prediction was significantly more accurate than random choice (Table 2). In addition, rules assuming directional preference (for the largest male) or homotypic preference (for a male of similar size as the female) led to less accurate predictions, which were not statistically different from random choice (Table 2).

**Table 2:** Prediction rate of the female choice as a function of the assumed preference rules

	**Prediction rate**		
	**Male 1**	**Linear order**	**Pairwise comparison**
Random choice	33%	17%	50%
Directional preference	36% [19%-50%]	28% [14%-42%]	53% [41%-64%]
Homotypic preference	36% [19%-50%]	25% [11%-39%]	61% [50%-70%]
Self-referent preference	67% [52%-76%]	42% [25%-58%]	78% [72%-84%]

Accurate prediction rate (with bootstrapped 95% CI in square brackets) of the most preferred male (male 1), linear order of the three males (male 1 > male 2 > male 3), and choice between two males in pairwise comparison as a function of the assumed preference rules: directional preference for the largest male, homotypic preference for a male of similar size to the female, or self-referent directional preference for males 30% larger than the female (Male/Female size ratio λ = 1.3).

## Discussion

The present study constitutes the first evidence for strong transitivity in a mate choice context. Evidence for SST suggests not only that female convict cichlids showed ordinal preference (as required under WST), but also that the direction and magnitude of their preferences between dyads of males could be predicted from their relative scaled values, as the difference between WST and SST is qualitatively similar to the difference between an ordinal and an interval scale [26]. Note however that the consistency of preference over time of transitive and intransitive females was not examined in the present study. It would therefore be valuable to assess in the future to what extent transitivity in mate choice is a characteristic of populations, or varies between individuals, possibly in relation to variation in age or personality, as the latter has recently been found to affect the measurement of female mate choice in vertebrates [52].

Due to the scarcity of empirical studies, it is difficult to contrast this first result with those of other studies. Kirkpatrick et al. [12] found no evidence for transitive or intransitive mate choice in females Tungara frogs, *Physalaemus pustulosus*. On the other hand, strong transitivity in a non-sexual context has been reported in some species, *e.g.* the unicellular slime mould *Physarum polycephalum*[53], European starlings *Sturnus vulgaris*[19,20], and domestic hens *Gallus gallus domesticus*[26], whereas both honey bees, *Apis mellifera*[17], and gray jays *Perisoreus canadensis*[18] were found to make intransitive foraging choices. Multidimensional choice [54] is often expected to lead to intransitive choice [1-3], unless animals are able to combine several criteria in a single common currency for fitness [46,55-57]. Our results thus suggest that male size could be an integrative criterion by which female convict cichlids assess the quality of potential mates. Indeed, body size of male convict cichlids has been found to correlate with several other traits such as aggressiveness [58], fighting ability [59], level of parental care [35], or nest site quality [60]. Although other male phenotypic attributes might be indicative of their quality, they may not necessarily be correlated with each other, and, hence, could be extremely complex to process simultaneously [61,62]. When faced with limited time and computational ability, animals are expected to rely on rules of thumb [63-65] such as fast and frugal heuristics that economize on computation by focusing on a limited number of cues [66]. Indeed, body size has been found to be the main male trait influencing female choice in several fish species [67,68]. Although it might be difficult to assess small size differences precisely [69,70], male body size should be quicker and easier to assess reliably than, for instance, level of paternal care or compatibility between mates [71,72].

Furthermore, our results strongly suggest that female convict cichlids do not use male body size as an absolute criterion, but, instead, as a relative one. Overall, females showed a consistent directional preference for males larger than themselves, but did not systematically prefer the largest male in a triplet. Instead, individual preference function reached a maximum for males approximately one third larger than the female (λ = 1.3). This is particularly interesting as much of the published evidence for female choice based on male size in convict cichlids remains inconclusive. For instance, using similar designs, some studies reported a preference for the larger of two males [30,42] whereas some others found no such an effect [73]. These results may not be as inconsistent as they appear if one considers that a female's preference for male size is proportional to her own size. Several studies on convict cichlids have shown that females benefit from mating with a larger male, in terms of breeding success [34] and protection of offspring against predators [35]. However, male preference for larger, more fecund females [39,74] and female-female competition may contribute to shape female preference for male size [33]. In the case of costly competition for mates, the poorest competitors may benefit from avoiding high-quality partners or targeting low-quality ones, in order to minimize costs [75-77]. This might be particularly relevant for convict cichlids, where a female's attractiveness to males has been found to be a function of her size relative to that of other females [74]. When mate choice is mutual, a female may thus save time and energy through directly attempting to mate with an individual of rank (in quality) similar to herself. In that respect, it is important to note that a self-referent directional preference (SRDP) for size is equivalent to a homotypic preference for rank in quality, if ranking in quality according to size does not differ between sexes (which is the case in cichlid fish where social dominance is linearly related to body size in both males and females [41]), and if the coefficient of SRDP precisely matches the degree of sexual dimorphism on the trait. Interestingly, the λ value leading to the most accurate predictions of choice in the present study, ranging from 1.29 to 1.32, is closely related to the level of sexual dimorphism observed in natural populations, where mean male length is about 30% larger than mean female length [30]. The λ value is also consistent with the size ratio (ranging from 1.27 to 1.35) observed within paired males and females [44,51,60]. In addition, any rule of thumb used in mating decisions should be flexible enough to accommodate changes in the social status of the animal through time, particularly when social status is directly related to size, and maximum size is not reached at sexual maturity. A SRDP for size might then be particularly relevant in species with mutual mate choice and continuous growth, such as monogamous fish, when individual quality is closely related to body size in both sexes.

A SRDP based on female size was, however, able to explain only four (L > M > S, M > L > S, M > S > L and S > M > L) out of the six transitive orderings that were observed in the present study. Four females showed transitive ordering that are not consistent with a SRDP: L > S > M and S > L > M. One possibility is that females might be subject to errors in their decision making [47,78-80]. It is also possible that a few females did not rely only on male body size relative to their own, but considered additional cues to make a choice [54,55]. If these cues vary independently or are only weakly correlated with size, they might explain the observed deviations from the four orderings predicted by the SRDP based on size only, including intransitive orderings [47,62,81].

Experimental evidence for transitivity in female convict cichlid preference may suggest that mating patterns observed in the wild result from rational mate choice. Mate preference, however, must be kept distinct from mate choice [82-84], as the preference relates to the sensory and behavioural components underlying female's willingness to mate with given male phenotypes, whereas mate choice results from the interaction between preference and external elements such as male availability, intra-sexual competition [33,68,85], and the cost of choosing [57]. On the other hand, our finding might be important to understand the dynamics of size-assortative mating in natural populations. Size-assortative mating is a pervasive pattern of mating in natural populations [86], particularly in monogamous fish species [87-89], including convict cichlids [74,90-92]. Although size-assortative mating can be produced independently of active mate choice, either through heterogeneity in spatial [93] or temporal [94] distribution of potential mates or through intrasexual competition [85], active mate choice may contribute to generate the observed patterns of size assortment between mates [88,95]. Still, the exact nature of the preference underlying observed patterns of size-assortative mating often remains unidentified [94,96]. In particular, patterns of phenotypic assortment can correspond to either a homotypic preference (or preference for one’s own type, [86,97,98]) or a directional one ([86]; or type preference [97,99]), or a combination of both at the population level. In practice, however, it is difficult to infer the individual process of mating from the mere consideration of mating patterns at population level [85,94,97,100]. Our results suggest that examining transitivity in mate choice in both males and females may help to understand how assortative mating occurs in natural populations.

## Conclusion

In summary, we performed an experimental study investigating the transitivity of choice, one key component of rationality, in a sexual context. Several authors have proposed that choice between partners might be intransitive [1,2,12]. This study provides the first evidence for strong stochastic transitivity in a context of mate choice. The females exhibited an ordinal preference, and the direction and magnitudes of this preference could be predicted from a simple comparative rule. We argue that the females did not necessarily compare two males to choose the best one. It is sufficient to assume that they used a self-referent evaluation based on their own size: females preferred male about one third larger than themselves. Such a decision rule has important implications for the evolution of the mate sampling strategies as it is less cognitively demanding than the comparative evaluation of the value of several males encountered sequentially. Several species have been reported to be able of self body-size perception [101]. In addition, they can constantly update the estimation of their physical characteristics, which is of crucial importance in case of continuous growth. Perception of spatial layout could be achieved when interacting with the environment, for instance, when the individual pass through various size apertures [102,103]. Social experience and direct interaction with conspecifics may also be involved in the constant reassessment of their physical ability or ressource holding potentiel [104-106]. Females Convict cichlid can use their body length as a template, and, importantly, may not have to learn and adjust their decision criterion across their lifetime as their template would systematically increase with their size. Finally, this decision criterion is consistent with patterns of assortative mating repeatedly observed at the population level.

## Materials and methods

### Biological material

One hundred convict cichlids (40 females, 60 males) were purchased from local commercial distributors. In the laboratory, they were maintained in 90 to 250 L tanks filled with water which was aerated and chemically and biologically filtered, at 25 ± 1°C under a 12:12-h light:dark cycle. Fish were fed twice a day to satiation with Tetramin cichlid flakes. The sexes were kept separately for two months before the experiments started in order to control for previous breeding experience and ensure sexual receptivity. Prior to the experiments, we formed 20 pairs of females, consisting of one small and one large female with a difference of at least 1 cm between them (Figure 1), and 20 triplets of males, consisting of one small male (S), one medium size male (M) and one large male (L) with a difference of at least 1 cm between each individual (Figure 1). We then randomly assigned each triplet of males to one of the female pairs. This paired design was adopted to ensure that potential differences in behavior between females of different sizes would not be due to any differences between the males they faced. All individuals were sexually mature at the time of the experiment.

### Experimental procedure

In order to assess transitivity in choice, each female was tested on three separate occasions in a two-way choice apparatus consisting of a 90 L aquarium partitioned into three sections with two leakproof glass partitions [30,42]. The left and right end sections each contained one male, while the central compartment contained the female. The central compartment was delineated into three areas (the left choice area in front of the left male, the central, or neutral, area, and the right choice area in front of the right male), using two opaque plastic partitions. These partitions were placed in the female’s compartment in such a way that the female could freely swim from one side of the compartment to the other without being able to see both males at the same time [30]. These partitions removed any effect of male–male competition through preventing visual interactions between them. The aquarium was lit by one daylight neon lamp tube (Sylvania Aquastar 30 W, 10000 °K) situated 10 cm above water level, while the room was maintained in the dark.

All individuals experienced only one test per day in order to limit contrast effects between trials (thus satisfying the assumption of independent and identically distributed random variables, [16]). Before each test, a 22 h-period of acclimatization took place, during which females were separated from males by opaque dividers. Positions of the males in the choice apparatus were randomized to avoid any bias associated with a systematic female preference for one side of the aquarium. In addition, the order of presentation of the different male dyads (L *vs.* M, M *vs*. S, and L *v*s. S) was randomized across tests to avoid primacy or recency effects [84,107]. Each test then lasted for two hours, during which time the female’s position was continuously recorded with a digital video camera (JVC Everio GZMG21) positioned in front of the aquarium. The study was carried out in accordance with the ethical standards of the the French National Centre for Scientific Research and was approved by the government authorities (permit n° 21-CC-EL-21).

### Transitivity

We assessed both WST and SST from the preference expressed by females in the choice apparatus. A female was assumed to prefer male A over male B when the preference index (amount of time spent in front of male A divided by the total amount of time spent in the two choice areas) was above 50%. With three alternatives (L, M, S), there are six possible transitive triads (L > M > S, L > S > M, M > L > S, M > S > L, S > L > M, and S > M > L), while non-transitive orderings can only occur with either L > M > S > L or S > M > L > S circular triads [108]. We assessed the degree of weak stochastic intransitivity as the proportion of circular triads [14]. We considered that the females made transitive choices if transitive ordering occurred more often than expected by chance (Binomial test with expected proportion of 6 transitive choices out of 8). For each female having made transitive choices (WST), we could then order the three males by order of decreasing female preference, from the most preferred male (male 1) to the least preferred one (male 3). Under the assumption of SST, the preference for male 1 when opposed to male 3 was expected to be higher than both the preference for male 1 when opposed to male 2 and that for male 2 when opposed to male 3.

### Mate choice rule

In order to assess which rule was used by females, we considered several candidate rules – namely a directional preference for the largest male, a homotypic preference for a male of similar size to the female, or a self-referent directional preference – and compared their ability to predict the female’s choice. In the later rule, the preferred male size was assumed to correspond to a fixed ratio to that of the female. For a given female of size *S*, we assumed that her ideal male size would be equal to λ×*S*, where λ denotes the value of the ideal ratio [68]. We then ranked the three males presented to the female according to their absolute difference with the predicted ideal male size, with the predicted preferred male being the individual whose size would be the closest to (*λ*×*S*), and so on. For each rule, we calculated the proportion of accurate predictions for the most preferred male (male 1), the linear order of the three males (male 1 > male 2 > male 3), or the preferred male in each pairwise comparison. Non-parametric bootstrap estimates were computed to assess the statistical confidence of predicted choice outcomes and compare prediction rate between rules [50].

### Statistical analysis

Data were inspected for homoscedasticity using the Brown–Forsythe test, and for normality using Shapiro-Wilk test, prior to the use of parametric tests. Proportions were normalized using square root-arcsine transformation. Differences in preference index between trials, and differences in male size as a function of their rank were analyzed using mixed-effect models including random slope to take into account repeated measures from the same females [109], using *nlme* package in R. Tukey’s *post-hoc* comparisons were performed using *glht* package. The Pearson correlation coefficient between female size and that of her most preferred male (male 1) was used as a measure of assortative mating [110]. All the tests were performed using R 3.0 software [111].

## Competing financial interests

The authors declare that they have no competing interests.

## Authors’ contributions

F-XDM, MF and FC designed research, F-XDM, MF, SM performed research, F-XDM and FC analysed the data, F-XDM and FC wrote the paper. All authors read and approved the final manuscript.

## Supplementary Material

Additional file 1 Supplementary materials. Click here for file
