# Supplementary material for: Female mate choice in convict cichlids is transitive and consistent with a self-referent directional preference
Source: Front Zool. 2013 Nov 11;10:69. doi: 10.1186/1742-9994-10-69 (PMC3828482; doi:10.1186/1742-9994-10-69)
Supplement: Additional file 1 — Supplementary materials. [file 1742-9994-10-69-S1.pdf]

Female mate choice in convict cichlids is transitive and consistent with a self-referent directional preference. François-Xavier Dechaume-Moncharmont, Marine Freychet, Sébastien Motreuil, Frank Cézilly.

Supplementary materials

Stricter preference criterion. In the main text of the article, we presented the analyses based on the complete dataset (sample size n = 40 females). We considered that a female preferred the male in front of which she spent more than 50% of her total time in the choice areas. This procedure has been previously shown to correlate with the final reproductive preference measured as the spawning behavior [1]. The proportion of time spent in front of the preferred male was generally high (mean preference index = 71.5%, 95% CI from 68% to 74%). However, in 17 out of 120 trials, the females spent less than 55% in front of their preferred male. One could wonder if a female spending, for instance, 52% of her time with male A is really demonstrating a preference for that male. We investigated whether a stricter preference criterion would modify our results. By discarding every female that exhibited at least one preference index lower than 55%, we considerably reduced the sample size down to 24 females, ignoring 55% of the females that were initially used in the experiment.

Using the reduced dataset with only the females having spent more than 55% of their time in front of the preferred male in each of their three trials (sample size n = 24 females), we repeated every statistical analyses. The new results were consistent with those reported in the article. We observed two intransitive females, which was again significantly more frequent than expected by chance (exact binomial test B(24, 0.75), p

= 0.040). We still conclude to the strong transitivity: the preference indexes of the 22 transitive females differed between the three dyads of males (Fig. S2, $F = 6.90$, df = 2 and 42, $p = 0.0026$). Mean preference index was significantly higher when the females had to choose between males of distant ranks (1 vs. 3), i.e. their most preferred and least preferred males, compared to when choosing between males of adjacent ranks (1 vs. 2: $p = 0.0014$, 2 vs. 3: $p = 0.033$), whereas the preference index when choosing between either male 1 vs. male 2 did not differ from that when choosing between male 2 vs. male 3 ($p = 0.35$). Mean male size decreased with rank preference for small females (Fig S3.A, $F = 5.81$, df = 2 and 20, $p = 0.010$), whereas it increased for large ones (Fig S3.B, $F = 15.1496$, df = 2 and 20, $p = 0.0001$). Because of a smaller sample size, the most accurate predictions of the preferred male were achieved for a wider range of λ values than the one reported with the complete dataset. The pattern is yet qualitatively preserved (Fig. S4).

Reference

1. Dechaume-Moncharmont F-X, Cornuau JH, Keddar I, Ihle M, Motreuil S, Cézilly F: **Rapid assessment of female preference for male size predicts subsequent choice of spawning partner in a socially monogamous cichlid fish**. *C R Biol* 2011, 334:906–910.

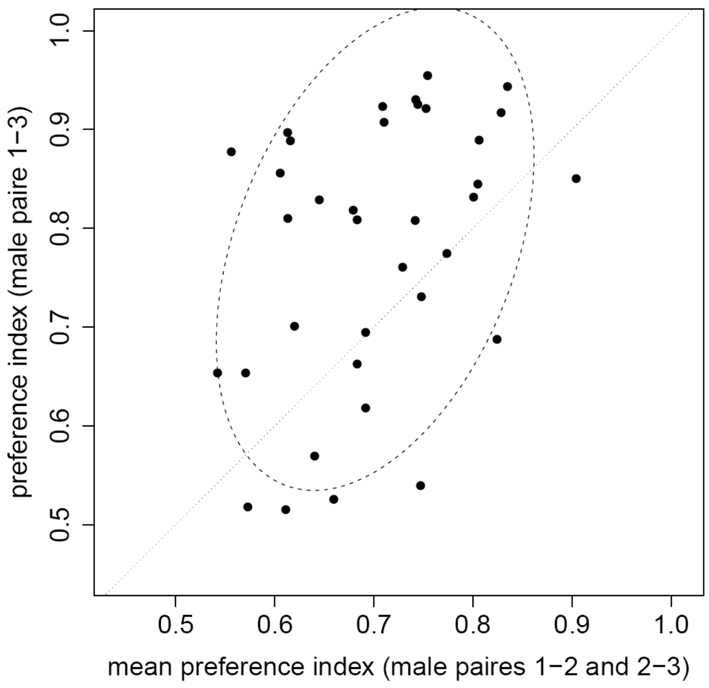

Figure S1 – Correlation between the preference index when choosing between the most extreme males, i.e. the most preferred male (male 1) vs the least preferred male (male 3) and the mean preference index when choosing between males of adjacent preference ranks, male 1 vs male 2 or between male 2 and male 3. Sample size is n = 36 females having made a transitive choice (male 1 was preferred over male 2, male 2 over male 3, and male 1 over male 3). The preference index is defined as the mean proportion of time spent on the side of the preferred male during a trial. Dotted ellipse figures the 95% confidence region for the bivariate-normal distribution to illustrate the correlation (Pearson correlation coefficient r = 0.39, p = 0.020). The dotted line figures the line of slope = 1.

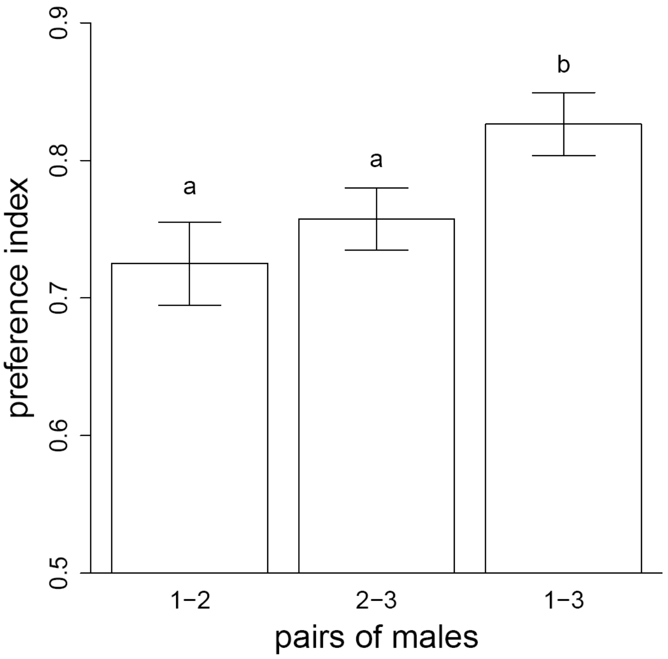

Figure S2 – This figure corresponds to Fig. 2 in the article. It was computed with a reduced dataset only including the transitive females that had preference indexes always larger than 0.55 (sample size n = 22). It shows the mean proportion of time (+/- standard error) spent on the side of the preferred male during each trial by the females having made a transitive choice (male 1 was preferred over male 2, male 2 over male 3, and male 1 over male 3). For example, the first bar depicts the proportion of time spent on the side of the male 1 when tested with the male 2. Different letters mean statistically significant differences.

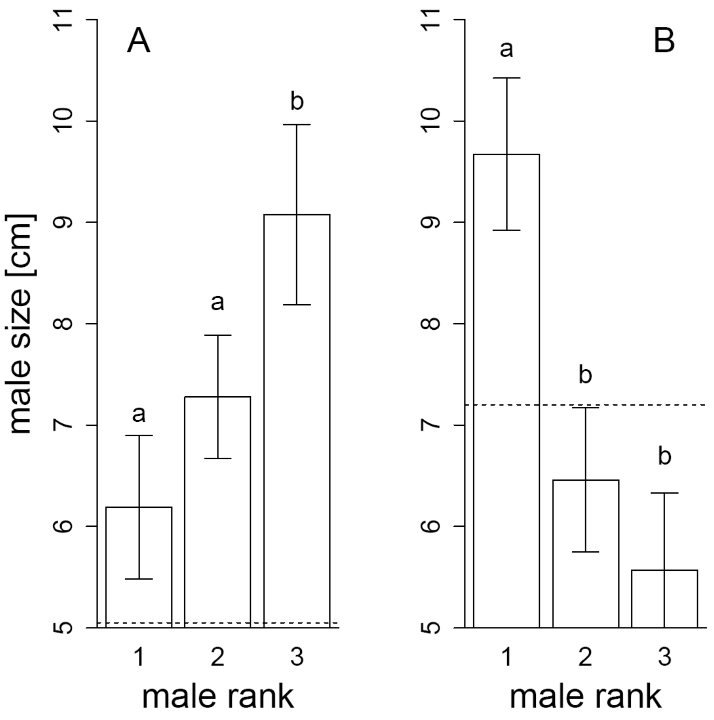

Figure S3 – This figure corresponds to Fig. 3 in the article. It was computed with a reduced dataset only including the transitive females that had preference indexes always larger than 0.55 (sample size n = 22). It shows the mean male standard length (+/- standard error) as a function of their rank by the transitive females: male 1 was preferred over male 2, male 2 over male 3, and male 1 over male 3. Different letters mean statistically significant difference. The horizontal dotted line depicts the mean female size. [A] Choice made by the smaller female (n = 11). [B] Choice made by the larger females (n = 11).

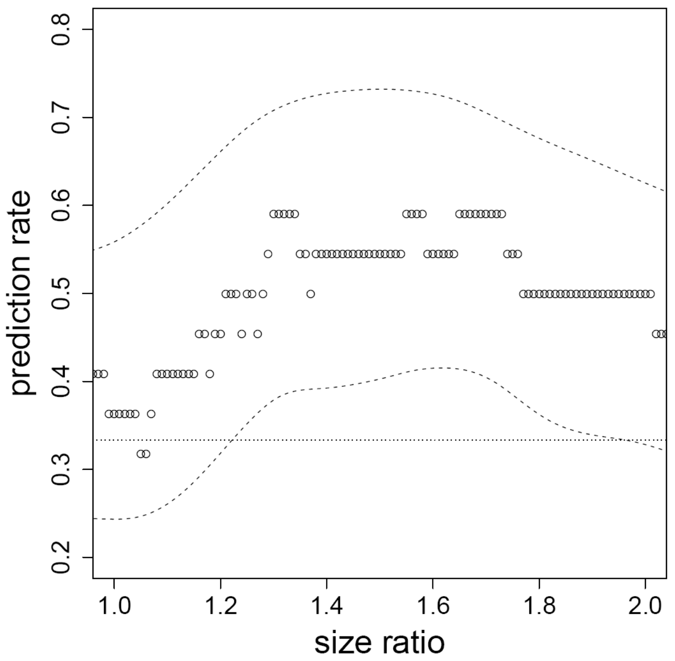

Figure S4 – This figure corresponds to Fig. 4 in the article. It was computed with a reduced dataset only including the transitive females that had preference indexes always larger than 0.55 (sample size n = 22). It shows the proportion of correct predictions of the preferred male as a function of the ideal ratio λ = male size / female size. For a given ratio, a female was assumed to prefer the male leading to the ratio that was the closest to the ideal one. The dashed curves depict the bootstrap 95% confidence interval around the prediction. The horizontal dotted line depicts random choice (one in three chance of choosing the preferred male). Preference is then inferred from the relative amount of time spent next to each male. A female is thus assumed to prefer male A over male B (A>B) when the amount of time spent in front of male A divided by the total amount of time spent in the two choice areas, considered hereafter as the preference index, is above 50%.

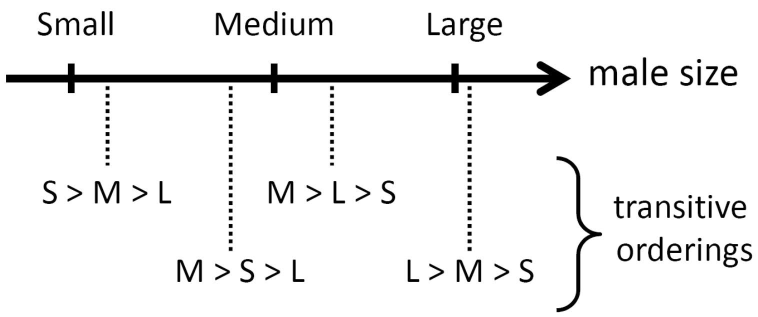

Figure S5 – Illustration of the four possible transitive orderings predicted by the self-referent directional preference (SRDP) based on the female own size. The three males are ranked according to their body length: small, medium or large size. The vertical dotted lines show several examples of ideal male sizes, which are function of the female's size. According to their distance to this ideal male's size, the three males are more or less attractive for the corresponding female. The SRDP based on the female own size is potentially able to explain only four (L>M>S, M>L>S, M>S>L and S>M>L) of the six observed orderings. The ordering of the three categories of male cannot be modified: by definition, the small male is always smaller than the medium, and so on. The SRDP is thus unable to explain two transitive orderings (L>S>M and S>L>M) that were observed in four females.
